# Emerging roles for AQP in mammalian extracellular vesicles

**DOI:** 10.1016/j.bbamem.2021.183826

**Published:** 2022-03-01

**Authors:** Charlotte E. Clarke-Bland, Roslyn M. Bill, Andrew Devitt

**Affiliations:** College of Health and Life Sciences, Aston University, Aston Triangle, Birmingham B4 7ET, UK

**Keywords:** Aquaporin, Water channel, Extracellular vesicle, Exosome, Microvesicle, Signalling, Oedema, Inflammation

## Abstract

Recent research in the aquaporin (AQP) field has identified a role for diverse AQPs in extracellular vesicles (EV). Though still in its infancy, there is a growing body of knowledge in the area; AQPs in EV have been suggested as biomarkers for disease, as drug targets and show potential as therapeutics. To advance further in this field, AQPs in EV must be better understood. Here we summarize current knowledge of the presence and function of AQPs in EV and hypothesise their roles in health and disease.

## Highlights

• Extracellular vesicles (EV) deliver cargo from donor to recipient cells, modulating physiology
• Aquaporins (AQP) are components of EV in both healthy and diseased states and can be isolated from body fluids
• Potential functional roles of EV-bound AQP in donor and recipient cells and in EV themselves are discussed.

## Graphical abstract

**Figure:**
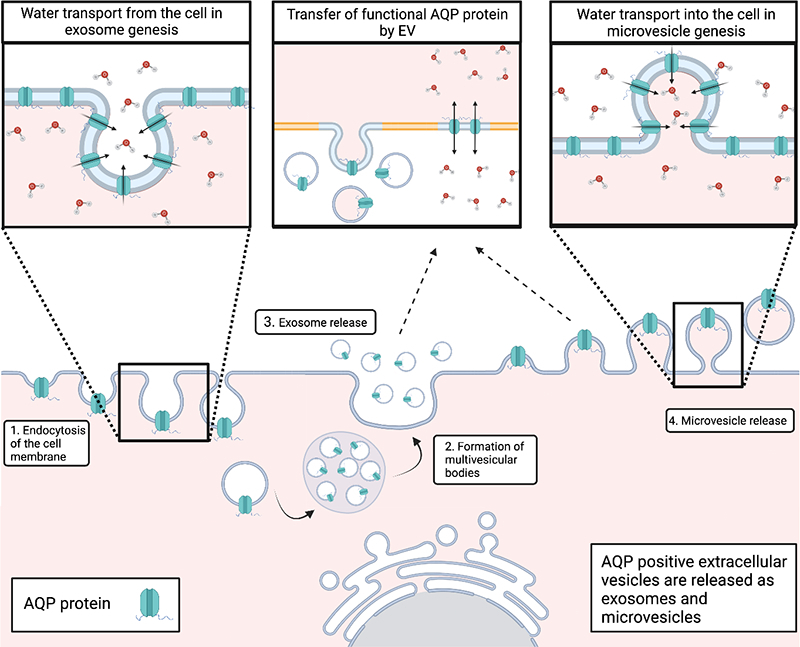

## 1 Introduction

**Fig. 1:**
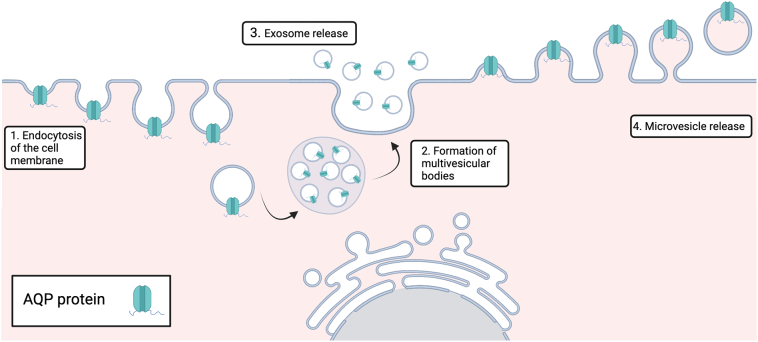
Extracellular vesicle formation. Step 1. Exosomes are product of endocytosis. The plasma membrane invaginates to form intracellular vesicles. Step 2. These vesicles form multivesicular bodies (MVBs). Step 3. MVBs fuse with the plasma membrane to release these vesicles as exosomes. Microvesicles are EV derived directly from the plasma membrane as it pinches off into the extracellular space.

Extracellular vesicles (EV) are naturally occurring nano or micro sized particles [1] released by cells into their extracellular environment [2] EV are representative of the properties and state of their donor cell, from which they are derived, carrying cell cargo such as functional nucleic acids, proteins, and lipid-based components [3], [4]. They play roles in a wide range of processes such as the transport of enzymes [5], micro RNAs [6], antigens and membrane proteins [7]. Two main classes of EV are widely accepted based on their site of subcellular origin. Whilst naming within the field is often varied, these classes are most commonly named exosomes and microvesicles/microparticles (Fig. 1). Exosomes are defined as being small in size, 30–150 nm, and derive from cytoplasmic multivesicular bodies, made via the endosomal route. They are characterised by the presence of ESCRT proteins such as TSG101, VPS4 and Alix [8] Microvesicles bud directly from the plasma membrane and are generally bigger than exosomes (100–2000 nm), though there is overlap in their size [9]. The origin of vesicle biogenesis is of interest as it can elude to the cargo and functions of the EV. Both healthy and diseased cells release EV into their extracellular environment, though the EV cargo can differ in these different states. For example, EV released by stressed or activated cells can contain pro- or anti-inflammatory molecules. Whilst EV may be implicated in waste disposal, it is also thought that they play a major and expanding role in cell communication, carrying information about the health or otherwise of the donor cell from which they are released; as such EV have been identified as biomarkers for disease. They can be isolated from bodily fluids such as cerebrospinal fluid, saliva, breast milk, blood, amniotic fluid, menstrual fluid and semen, and some can be used as a liquid biopsy with minimal invasion, especially when compared to alternatives such as tissue biopsy. Exogenous EV also show potential as therapeutics, delivering functional cargo where endogenous versions are absent or defective.

Aquaporin water channels (AQP) form a family of small (~30 kDa), integral, transmembrane channel proteins [10]. They reside in the plasma membrane of cells [11] where they form pores, facilitating the rapid, passive and bi-directional movement of water, small neutral solutes and some ions into and out of cells in response to osmotic or concentration gradients [12]. Our understanding of AQPs has expanded considerably, since their discovery in 1992; the structural, functional, homeostatic and pathophysiological roles of each mammalian AQP have been described to some extent. They have been identified in all kingdoms of life and 13 mammalian AQPs (AQPs 0–12) have been identified thus far.

AQPs comprise six transmembrane helices, intracellular N- and C- termini and five loop domains. The family's signature NPA (asparagine-proline-alanine) motif defines their selectivity to water and variations in this domain give rise to differential permeability profiles and subclasses of AQPs [13] Orthodox AQPs (0,1,2,4,5,6 and 10) are permeable to water (and some also act as ion channels), aquaglyceroporins (AQPs 3, 7 and 9) are permeable to glycerol, urea and some other solutes, whilst super or unorthodox aquaporins (AQPs 11 and 12) are the most recently and incompletely defined family members (AQP8 is an ammonia- permeable AQP related to the orthodox sub-group [13]).

AQPs can also show different intracellular localisation; though they are primarily located in the plasma membrane, some are also expressed within intracellular vesicles and traffic to the plasma membrane in response to external stimuli including hormones, hypotonicity and hypoxia [14], [15], [16], [17]. AQPs have been identified in nuclear membranes.

In vivo, AQPs predominantly form homotetramers, with each AQP monomer possessing a functional, water permeable, central pore [18] AQPs are ubiquitous in the human body and have been implicated in a broad range of pathologies. For example, nephrogenic diabetes insipidus is characterised by an inability to concentrate urine, and has been linked to mutations of AQP2 that greatly impede water channel function [19]. AQP4 is associated with the development and resolution of brain oedema following injury and stroke [20], [21] Alternative roles for AQPs have also been suggested due to their association with conditions apparently unrelated to water transport; AQP negative cancer cells have decreased motility, suggesting a role for AQPs in cell migration [22]. AQPs also contribute to cell membrane integrity [23] and cell communication [24].

To date the mammalian AQPs 1, 2, 3, 4, 5, 7 and 9 have been found in association with EV (Table 1, Table 2, Table 3), in a wide variety of organisms and cell types ([53], http://microvesicles.org/) suggesting AQPs in EV may have diverse roles. Studies have shown differences in the cargo of exosomes compared with microvesicles derived from cells, exosomes are created by invagination into the cell and formation of the endosome and interaction with the endoplasmic reticulum, whereas microvesicles pinch directly off the plasma membrane. Differences in cargo include changes in the orientation of membrane proteins, where proteins in EV are ‘intracellular side out’ [54]. A current topic of interest in the AQP field includes their presence and functional significance in EV, as they are likely to modulate the EV environment and are also taken up and integrated into nearby cells [55]. In the case of AQPs, this would potentially mean exposure of intracellular N- and C-termini and intracellular loops to the extracellular space [56]. As these regions of AQPs are key areas for phosphorylation and protein interaction this is of particular interest in AQP-EV discussion (Fig. 1).

**Table 2:** Studies focussed on CNS AQP-EV published to date. These studies are based on data from CSF and blood samples AQP- EV have been identified in diseased patients and animal models. Some studies report the size and type of vesicle containing AQPs. Publications have been organised in the order, they are discussed in this review. Studies discuss EV using a variety of terminology as shown (e.g. EVs, exosomes, microparticles) depending on the focus of the study and findings; within the body of this review, all are referred to as EV.

System	Aquaporin/s identified	EV terminology	Pathology/treatment	Experimental model	Authors
CNS	4	Microparticles	Traumatic brain injury	Human	[45]
	4	Microparticles	Neuromyelitis optica spectrum disorder	Human	[46]
	4	EV	Alzheimer's disease	Mouse	[47]
	4	Exosomes	Alzheimer's disease	Rat	[48]
	4	EV	Stress-induced exhaustion disorder	Human	[49]

**Table 3:** All studies focussed on AQP-EV, other than urinary and CNS, published to date. These studies are based on data from saliva, blood and cells in vitro. These studies are described in the following paragraphs.

System	Aquaporin/s identified	EV terminology	Pathology/treatment	Experimental model	Authors
Digestive - saliva	5	Exosomes	Healthy	Human	[50]
Circulatory - blood	1	Exosomes	Healthy - maturing	Mice	[51]
Bone marrow	1	EV	Healthy	Human mesenchymal stem cells – in vitro	[52]

### 1.1 AQP in EV –studies to date

**Table 1:** Studies focussed on AQP-EV published to date. Most studies have used urine samples and studied renal AQPs. CNS studies are based on data from CSF and blood samples. Some studies report the size/density/type of vesicle containing AQPs. AQP EV have been identified in both healthy and diseased patients or animal models and have been isolated from in vitro mammalian cell supernatants. Publications have been organised by system and in the order they are discussed in this review. Studies discuss EV using a variety of terminology as shown (e.g. uEVs, exosomes, microparticles) depending on the focus of the study and findings; within the body of this review all are discussed as EV.

System	Aquaporin/s identified	EV terminology	Pathology/treatment	Experimental model	Authors
Urinary	2	Low density exosomes	Healthy	Human	[25]
	2	Exosome-like	Hepatic disease	Mouse & rat	[26]
	1, 2 & 7	Exosomes	Healthy	Human	[28]
	1 & 2	Exosomes	Healthy	Human	[29]
	2	Exosomes	Healthy	Human	[31]
	1 & 2	Exosomes	Healthy	Human	[32]
	2	Exosomes	Healthy	Human	[33]
	2	Exosomes	Desmopressin treatment	Human cell line	[35]
	1 & 2	uEVs	Acute Kidney Injury	Rat	[36]
	1	Exosomes	Acute Kidney Injury	Rat	[37]
	1	Exosomes	Pelviureteric junction obstruction	Human	[38]
	1 & 2	Exosomes	Transplant	Human	[39]
	2	Exosomes	Nephrosis	Human	[40]
	2	uEVs	Gentamycin treatment	Human	[41]
	2	uEVs	American cutaneous leishmaniasis	Human	[42]
	2	Low density	Autosomal dominant polycystic kidney disease	Mouse	[43]
	2	uEVs	Nephronophthisis	Mouse	[44]

## 2 AQP-EV in the urinary system

**Fig. 2:**
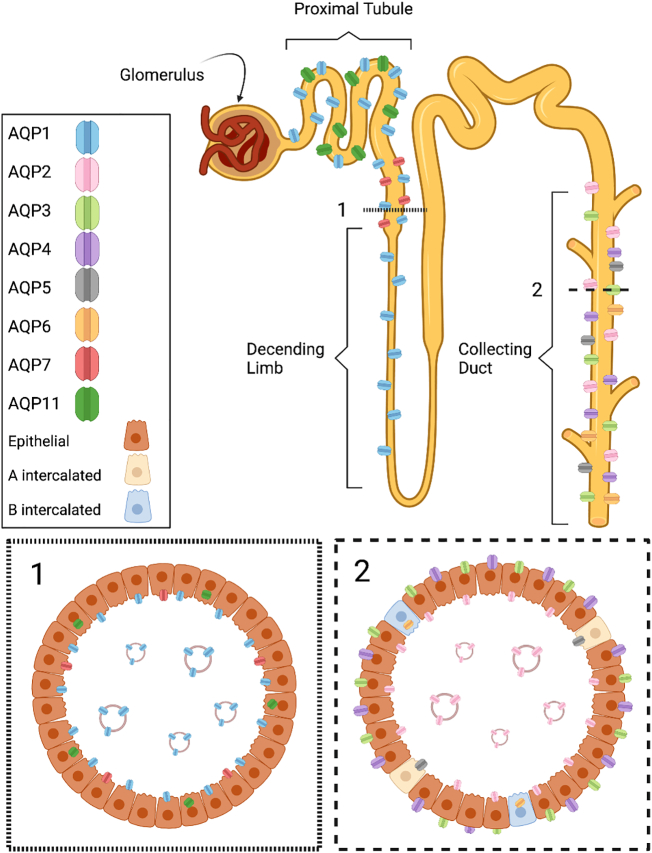
Nephronal, cellular and vesicular localisation of renal AQPs. Panels 1 and 2 represent transverse cross sections of the nephron in the loop of Henle (1) and the collecting duct (2). AQP1 is expressed in the descending limb of the loop of Henle; it is found in the apical membrane of epithelial cells and has been identified in uEV. AQP2 is located in the apical membrane of epithelial cells in the collecting duct and has been identified in uEV. AQPs 3 and 4 are located in the basolateral membrane of epithelial cells in the collecting duct. AQP5 is located in the apical membrane of intercalated A cells, whilst APQ6 is located in the cytoplasm of intercalated B cells. AQP7 is located in the apical membrane of epithelial cells in the distal convoluted where it acts primarily as a glycerol transporter. AQP 11 is expressed intracellularly, in the endoplasmic reticulum of epithelial cells in the collecting duct. Apical AQPS 1and 2 have been identified in urinary uEV.

AQPs 1–7 and 11 have been isolated from mammalian kidneys and are well characterised in terms of structure, function, localisation within the kidney, nephron and in subcellular regions (Fig. 2). Each renal AQP has a unique expression profile, with differential nephronal localisation directly relating to its role in water clearance and/or reabsorption [57]. The roles and expression levels of renal AQPs have been established in many pathologies with some considered targets for drug delivery.

It has been known for some time that kidney-derived AQPs are found in the urine of healthy patients [58] and changes in urinary AQP concentration are seen in both renal and systemic pathologies [59] suggesting that the presence and in concentration of urinary AQPs is a biomarker of disease. As AQPs are hydrophobic membrane proteins and their tertiary structure depends on their lipid bilayer environment, it is reasonable to assume that urinary AQPs are contained within a lipid bilayer and so, more recently, renal AQP research has included the role of AQPs in urinary extracellular vesicles (uEV), especially in renal and water balance pathologies [25], [26], [28], [29], [30]. uEV from urine samples account for the majority of published studies in AQP-EV research. To date AQPs 1 and 2 have been identified in uEV.

AQP2 is currently the most well researched renal AQP; it has been identified in pathologies such as renal injury, diabetes, cancer and other renal disorders [25], [31], [62]. Studies suggest a strong correlation between AQP2 presence and function in the kidney and in uEV. As such urinary AQP2 uEV have been suggested as a biomarker for a number of pathologies.

Studies that identify AQP2- uEV often show evidence that supports they are exosomes, generated via the endosomal pathway. They appear small, <100 nm in diameter [33]. AQP2 has been shown to function as a water channel in uEV allowing movement of water out of EV in response to osmotic challenge, measured by EV volume. In the presence of the AQP inhibitor mercury chloride, EV volumetric changes were not seen [33].

Renal AQPs facilitate the clearance and reabsorption of water by the kidneys, balancing overall water and ion levels in response to blood plasma osmolality [57]. Street et al. showed that AQP2 EV released from MCCDC11 stimulated with desmopressin are taken up by untreated AQP negative MCCDC11 cells which subsequently begin to express functional AQP2 [35]. The group identified AQP2 water channels in EV and speculate the transfer of functional proteins to neighbouring cells. They also speculated that EV might transfer AQP2 miRNA to neighbouring cells, resulting in AQP protein generation.

### 2.1 Acute Kidney Injury

Acute Kidney Injury (AKI) is a rapid onset impairment of kidney function which often occurs following ischemic reperfusion, when the kidney's blood supply has been limited due to a blockage, physical injury or reduced blood flow and the kidney has become ischemic. The tissue is injured further upon restoration of the renal blood flow and reoxygenation. AKI is characterised by tissue damage, inflammation, defective urine concentrating ability and dehydration. AKI is difficult to diagnose in its early stages due to a lack of AKI-specific biomarkers [63]. Differences in renal AQP expression levels in normal vs AKI tissue could help identify AKI in patients. AQPs 1, 2 expression is reduced in AKI rat models [36] and children with renal blockage injury show reduced expression of AQP1 [38]. Evidence suggests that AQP-uEV concentration in patient urine can be used as a biomarker to determine AKI severity; Sonoda et al. have identified changes in AQP1 concentration in uEV in AKI rats with early AKI, without reduced urine osmolality or increased urine volume. Bilateral AKI rats showed significantly reduced AQP1 uEV levels compared to those unilaterally injured suggesting AQP1 concentration per uEV reduces, rather than the number of uEV decreasing [37]. These data are supported by further studies; Asvapromtada et al. used a similar model and saw a reduction in both AQP1 and AQP2 concentration in uEV in AKI rats [36].

### 2.2 Transplant

Replacement of a defective kidney is sometimes the only option for treatment for severe disease or injury. The transplantation process faces complications of kidney injury and possible tissue rejection. Transplant patients must be closely monitored for kidney function. Following kidney transplantation patients suffer diuresis [64] and low urine osmolality suggesting a decrease in the function of renal AQPs 1 and 2. In a recent study by Oshikawa –Hori et al., levels of AQPs 1 and 2 were measured in the urine of both kidney transplant recipients and donors [39]. The group observed an initial decrease in uEV AQP1 & 2 expression one and two days after transplantation in both recipients and donors. This recovered to control levels by day six. Changes observed were significant for AQP2 but qualitative for AQP1 in the donor group. There was a correlation between urine osmolality and AQP2 uEV release where patients with more concentrated urine released more AQP2 positive uEV. This correlation was not observed for AQP1 uEV and urine concentration, the relationship between which appears to be unrelated [39]. This study suggests uEV AQP2 levels could be used as a biomarker for concentration defects within a week following transplantation as control levels for urine concentration and uEV-AQP1/2 expression is reached by day 6. Assuming each of the patients studied recovered well from the transplantation procedure, it could be that AQP2 could be a biomarker for renal defect following transplantation beyond 6 days in patients whose ability to concentrate urine and/or whose uEV AQP2 expression has not recovered by day 6 and beyond. This suggestion is supported by study of renal transplant rejection in rat models where whole kidney AQP2 expression is decreased on day 2 following transplantation and is down regulated by 40% by day 4 [165]. Similar results were observed by Chen et al. who also compared whole kidney AQP2 expression in a rat renal transplant rejection model but at 3, 5 and 7 days post transplantation [65].

### 2.3 Diabetic nephropathy

Diabetic Nephropathy (DN) is the gradual decline in renal function often seen in cases of both type 1 and 2 diabetes [66]. It occurs as a result of increased blood pressure and blood glucose levels and is characterised by high urinary glucose levels and polyuria. Patients with DN express renal AQP5 not normally found in healthy individuals. In vitro mouse models show AQP5 expression, co-localisation and direct interaction of AQP5 with AQP2, preventing its trafficking to the plasma membrane [67]. As AQP2 membrane localisation is essential for water reabsorption, it is reasonable to conclude that this AQP5 -related disruption of AQP2 membrane trafficking is responsible for the polyuria seen in patients with DN. Rood et al. have identified AQP2 in sub-populations of exosomes in human DN urine in high molecular weight samples but not lower. The group suggests AQP would be present in smaller EV also but predict their method for detection lacks sensitivity required for detection. They also note presence of high levels of soluble protein in samples that could interfere with their ability to detect proteins in samples. This study concludes that AQP2 is present in HMW particles and may or may not be present in smaller particles [40].

### 2.4 Drug toxicity damage

Many therapeutic drugs are nephrotoxic, causing tissue damage if used in high concentrations for long time periods. Abdeen et al. have shown that following treatment with the antibiotic gentamycin, kidney damage occurs and AQP2 uEV expression levels are altered, with increases or decreases seen at different timepoints [41]. They suggest AQP2 in uEV could be used as an early biomarker for drug- induced kidney damage.

### 2.5 American cutaneous leishmaniasis

American cutaneous leishmaniasis (ACL) is caused by the *Leishmania* parasite and is associated with reduced renal function. Oliviera et al. have demonstrated reduced renal AQP2 uEV in patients with ACL alongside reduced urinary concentrating capacity. This study suggests reduced renal AQP2 expression in line with previous studies, showing a correlation between uEV AQP2 expression and renal AQP2 expression levels [42].

### 2.6 Autosomal dominant polycystic kidney disease

In autosomal dominant polycystic kidney disease (ADPKD), renal cysts are formed. AQP1 expression was seen in within cysts and AQP1 expression decreased as ADPKD progressed and cyst size increased [68]. AQP2 has also been shown to play a role in the development of renal cysts, where overexpression of AQP2 promotes cyst formation [69]. This can be slowed by controlling and decreasing AQP2 expression [70]. In addition, AQP1 knock out mice are unable to traffic protein polycystin-1 (PC-1) to the plasma membrane effectively, resulting in the development of renal cysts [71]. It has been suggested that renal AQPs play an important role in the formation of renal cysts and renal AQP expression could be a targeted to prevent cyst growth. Hogan et al. were able to fractionate PKD uEV into sub populations; one of these was rich in AQP2. AQP2 was found in lowest density fractions [43]. This could suggest higher water permeability of AQP positive EV. Although this study does identify AQP2 presence and information about a potential AQP2 positive exosome subpopulation of low density vesicles, AQP2 is not a central focus of the study and actual densities for AQP2 exosomes are not provided. Nephronophthisis (NPHP) is a genetic disorder characterised by renal cysts. Symptoms include excessive thirst, polyuria with inflammation and fibrosis. Evidence shows increased AQP2 –EV levels in mice with NPHP up to 16 weeks of age when compared with control mice alongside polyuria [44].

The majority of studies on AQPs in uEV focus on renal AQPs 1 and 2; this is likely due to their important roles in water reabsorption into the bloodstream and their localisation to the apical membrane where they interface directly with urine. Work that seeks to identify EV subclasses of uEV suggests that AQPs are mainly found within exosomes, produced through the endosomal pathway. Given that AQP are known to traffic from intracellular vesicle ‘stores’ to the plasma membrane to enable the necessary rapid responses to changes in tonicity [14], [15] it is reasonable to assume that AQP would be present also in microvesicles, released directly from the plasma membrane. Our unpublished data confirm the release of EV in response to changes in extracellular hypotonicity, as seen in the kidney lumen. These EV are of varied size. It is also expected that AQPs 1 & 2 would be present in exosomes. It is widely known that AQP2 traffics to the plasma membrane from intracellular vesicles, so an association of AQP2 with MVB markers is to be expected. AQP1 has also been shown to traffic from intracellular vesicles in response to changes in extracellular hypotonicity [13], [14], [15], [72], [73].

There are many other renal AQPs yet to be identified in uEV. It is possible that there is selective loading of some AQPs to EV and this may be functionally significant. However, these AQPs may not be released in EV into urine, due to their subcellular localisations; in the kidney, AQP3 is localised to the basolateral membrane of principal cells in the collecting duct where it plays an important role in the concentration of urine [74] allowing movement of water into interstitial fluid. AQP4 is expressed alongside AQP3 in the basolateral membrane of collecting duct principal cells where it is essential for renal function [75]; AQP4 knockout mice have a reduced ability to concentrate urine [76], [77]. More recently, AQP5 has been identified in the human kidney. It is located exclusively in the apical membrane of type B intercalated cells in the collecting duct; the role of AQP5 in these cells is currently unknown. It has been shown to colocalize with Pendrin, an anion exchange protein [78]. It is expressed at low levels and localisation to the apical membrane of these cells. Procino et al. speculate a potential role for renal AQP5 as an osmoreceptor but no studies have evidenced this as yet. AQP6 is found exclusively in the cytoplasm of type A intercalated cells. It is different from other AQPs in that its permeability depends on the acidity of its environment [79]. AQP6 is permeable to glycerol, urea [80] and nitrate [81]. The exact role for AQP6 is not known but considering the differences between AQP6 and other AQPs, it is reasonable to presume that AQP6 plays a role different from that of a passive water channel. AQP7 is expressed in the apical membrane of the proximal straight tubules [82]. Evidence suggests that although AQP7 plays a contributing role in water reabsorption in the kidney, its primary function is the uptake of glycerol [83].

Studies that seek to identify renal AQPs 3, 4, and 11 in uEV have been unsuccessful [39]. This is not surprising as AQPs 3 and 4 are found in the basolateral membrane of polarised cells. If AQPs 3 & 4 EV are released in EV it would be into the surrounding interstitial fluid or neighbouring cells, perhaps playing a role in cell-cell communication. AQP5 is located in the apical membrane of intercalated cells, and intercalated cells have been shown to release EV, though AQP5 has not been identified in uEV. One possible explanation for this is that AQ5 is expressed only at low levels and current purification and detection methods may not be sophisticated enough to detect it. Alternatively AQP5 may not be present in uEV. AQP6 is an intracellular protein and no evidence suggests its trafficking to the plasma membrane so one would not expect to find AQP6 in uEV. AQP7 is located in the apical membrane of cells in the proximal tubule, is possible and has been identified in human urine [28]. It is interesting that the glycerol transporter AQP7 has not yet been identified in uEV. This could suggest that the presence of AQPs 1 & 2 in uEV is related to their water channel function and that uEV require water channels. Renal AQP11 is expressed in the endoplasmic reticulum and to our knowledge, there is no evidence to suggest its presence in exosomes or microvesicles. Fig. 2 presents the localisation or renal AQPs in the kidney and associated uEV as it currently known.

## 3 AQP-EV in the central nervous system

**Fig. 3:**
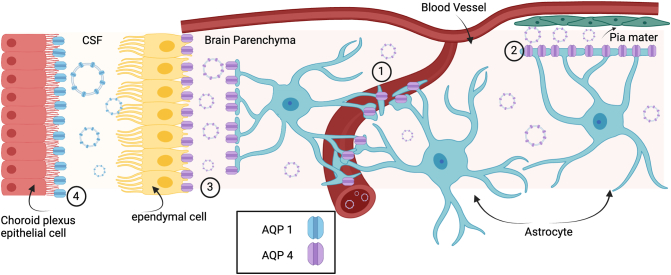
Localisation of CNS AQPs. AQP1 is located in the apical membrane of choroid plexus epithelial cells. AQP1 EV could be secreted into the CSF. AQP4 is expressed by astrocytes and can be found at interfaces with AQP4 in the basolateral membrane of ependymal cells, in perivascular endfeet and at the pia mater. AQP4 EV could be secreted into the interstitial fluid, blood and cerebral spinal fluid near the pia mater.

The Central Nervous System (CNS) comprises the brain and spinal cord. It is surrounded by cerebrospinal fluid and receives nutrients, oxygen and water from the blood via the blood brain barrier (BBB). CNS-derived EV (cnsEV) have been identified in cerebrospinal fluid and blood [84], [85] in both healthy and pathophysiological states, including brain tumour [86] stroke [87], and traumatic brain injury (TBI) [45]. CnsEV have also been identified as potential biomarkers for injury severity [88], [89], [90], [91]. AQPs 1 & 4 are expressed in the CNS and function primarily as water channels. AQP1 is expressed in the apical membrane of epithelial cells in the choroid plexus (Fig. 3) [92] and is responsible for water secretion into the CSF [93] AQP4 is the most abundant CNS AQP [94] It is expressed by astrocytes, star shaped glial cells that maintain brain homeostasis, communicating with and supporting neurons. Astrocytes are central in intercellular CNS communication and recent research has shown that astrocyte-derived EV contain functional proteins and are taken up by neighbouring cells [95].

Complications of many CNS pathologies include secondary injury caused by inflammation and/or swelling as a result of rapid and excessive accumulation of water in CNS tissue (oedema). As the most abundant water channel in the CNS, AQP4 has been implicated as the channel responsible for the movement in water in these pathologies. CnsEV have also been identified in CNS pathologies including, viral infection [96] prion disorders [97], neurodegeneration [48] multiple sclerosis [98] CNS cancer [99] lipid storage diseases and injury and infection. AQP positive cnsEV (AQP-cnsEV) have been identified in several of these pathologies though to date AQP-cnsEV studies have been less abundant than AQP-uEV; sample collection is more invasive for cerebrospinal fluid sampling over urine and blood samples are perhaps more difficult to process and less reliable due to the presence of serum vesicles and high concentrations of lipoproteins.

Pathologies associated with CNS tissue injury are usually associated with oedema. There are a number of CNS pathologies that cause CNS tissue to become injured. APQ4 has been shown to play a leading role in CNS oedema formation and resolution, allowing rapid movement of water into and out of the CNS. AQP4 and EV have been implicated in a number of CNS injury related pathologies as discussed below.

### 3.1 Stroke

During ischemic stroke, arterial blood supply is blocked and oxygen supply is compromised. This results in cell death and can cause brain damage and even death. Ischemic stroke is known to cause brain oedema. Manley et al. demonstrated that deletion of CNS AQP4 reduces brain oedema in mice following ischemic stroke when combined with water intoxication [100]. This suggests that AQP4 plays a role in oedema associated with ischemic stroke. Studies have identified EV release following stroke and hypothesise that these could play a protective role against further neuronal damage [101].

### 3.2 Traumatic brain injury

Traumatic brain injury (TBI) is either direct or indirect injury to the brain tissue and may result in a loss of BBB integrity, blood vessel damage, cell damage and death, inflammation and oedema [102]. Depending on the severity of injury, TBI can result in secondary injury which is associated with poor patient outcome, permanent brain damage and loss of life. AQP4 expression in the brain increases following injury and an increase in brain water content is observed [103]. In rats, AQP4 expression not only increases in the area of injured tissue but also in other, uninjured regions of the brain following focal injury [104]. Studies show that inhibiting AQP4 function reduces cerebral oedema following TBI, suggesting AQP4 as an important potential drug target [15]. Activated astrocytes and microglia release EV following TBI [105]. The contents of these ‘response’ EV are pro-inflammatory [106] and are thought to propagate damaging inflammation. Levels of these response EV may correlate with injury severity and therefore patient outcome highlighting the possible value of EV as biomarkers. EV containing AQP4 have been identified in the blood stream of patients following TBI has been implicated as a biomarker for severe TBI due to its increased levels in EV in TBI patients versus those without injury [45]. It has been shown that perivascular AQP4 expression increases following injury [15] this correlates with an increase in EV AQP4 concentration. It has also been suggested that the release of AQP4 into EV could protect astrocytes from further oedema.

Astrocytes that interface with blood vessels make up the BBB (Fig. 2). When astrocytic endfeet swell or these astrocytes die, the BBB is compromised and immune cells, previously unable to enter the CNS are able to infiltrate the space. Though immune cells aid in clearance of dead and dying cells, they also release reactive oxygen species (ROS), causing further damage, and recruit more immune cells. This propagation causes inflammation and causes secondary injury to tissue. As with AQP2 in the kidney, it may be possible that AQP4 is taken up and expressed by neighbouring cells. Studies that show AQP4 expression increases in areas of the tissue outside the injured region. This might suggest some kind of intercellular communication such as EV transferring AQP4 to other regions of the brain. Also, it may be that AQP4 plays a role in membrane integrity of cnsEV and thus extends the lifetime of circulating EV. It is known that following CNS injury, water/solute levels change. APQ4 may, like AQP2 in urine, allow cnsEV to adapt to osmolar changes in their environment, preventing excessive swelling or shrinkage.

### 3.3 Glioma

AQP4 expressing astrocytes, like other cells, are susceptible, to neoplastic change and the most common and malignant glial cancer is glioblastoma multiforme (GBM). Increased AQP4 expression has been seen in high grade tumours and a variety of roles suggested including water permeability [107] and glial cell migration, with AQP4 knockout glial cells having reduced migratory and invasion ability [108]. This suggests cerebral AQP4 could be a potential drug target in the treatment of glioma.

### 3.4 Neuromyelitis optica spectrum disorders

Neuromyelitis optica spectrum disorders (NMOSD) is an autoimmune disease characterised by brain lesions. Patients often show autoimmunity to AQP4, producing antibodies (NMO-Ab) that bind to the AQP4 protein [109], [110] inducing an inflammatory cascade response that leads to demyelination, cell damage and death. Encephalitis is also, but less commonly seen, in NMOSD. In vitro studies show there are likely multiple epitopes to which AQP4 autoantibodies bind, resulting in a variety of consequences such as endolysosomal degradation, breakdown of the BBB and changes in water transport via AQP4 [111]. Studies suggest that AQP4 may be internalised upon binding of NMO-Ab, initiating a complement cascade. It is possible that the internalisation of AQP4 reduces the inflammatory response and the associated negative consequences. AQP4 positive microparticles have been identified in patients with NMOSD. Bejerot et al. suggested AQP4 positive EV cause widespread inflammation and production of autoantibodies [46]. Lee et al. compared exosomes from the cerebrospinal fluid of NMOSD and multiple sclerosis patients and did not note any differences in AQP4 content [112]. Further study into the inflammatory properties of AQP4 positive, glial cell-derived EV could further our understanding of CNS autoimmune disease.

### 3.5 Alzheimer's disease

Alzheimer's disease is a form of dementia, characterised by β-amyloid plaques and tangles of the tau protein. It is well established that EV are released by cells of the CNS and it has been suggested that these EV contribute to both physiology and pathology within the CNS. EV have been reported within Alzheimer's disease, tau related pathology and dementia with Lewy bodies. Studies report that EV can transmit pathology, in support of a prion-like aetiology [113], [114]. The role of EV within neurodegeneration is complicated and, at times, paradoxical. In Alzheimer's disease, beta amyloid is released within EV from neurons [115]. This has been suggested as a ‘waste disposal’ mechanism [116] and these neuronal-derived EV have been proposed to reduce build-up of amyloid plaques [117], [118]. This suggests that EV may have a *neuro-protective* function associated with the transfer of amyloid beta to microglia. However, it is clear that not all EV function in a similar manner; whilst disaggregating beta amyloid, microglia release neurotoxic forms in EV; these microglia-derived EV promote a *neurotoxic* effect. EV are also known to be released from other cells of the neurovascular unit. Astrocytes release EV that contribute to glial cell death [28] but are also implicated in neuronal survival [119]. EV are also released from pericytes, a key cell type involved in the control of neurodegeneration [120]. AQP4 has also been linked to Alzheimer's disease pathogenesis and suggested as important in the clearance of beta amyloid and tau. Denver et al. found that in rats with Alzheimer's disease AQP4 polarisation to astrocytic endfeet is disrupted and expression is dispersed. In addition, AQP4 localisation in blood serum exosomes was increased significantly in AD rats [48]. This work is supported by recent findings using AD mice which also shed increased levels of AQP4 positive EV [47]. Further work is needed to understand whether this AQP4 release is part of a waste disposal or neuro protective function but given the role of AQP4 in modulating macrophage motility, it seems likely that AQP4 in EV have a functional significance yet to be determined.

### 3.6 Stress induced exhaustion disorder

Stress induced exhaustion disorder (SED) is also known as clinical burnout. Patients suffering with this condition experience chronic fatigue and cognitive dysfunction. SED patients show symptoms similar to those with TBI such as long-term impairment of cognitive function and memory. Wallensten et al. have demonstrated that SED patients have increased levels of circulating astrocyte- derived AQP4 positive EV that those with depressive disorders such as major depressive disorder. AQP4-EV levels in SED patients correlate more closely with those previously seen in TBI patients [49].

## 4 AQP-EV in reproductive systems

Aquaporins 1–12 have been identified in the female reproductive system of mammals including the ovary (AQPs 1,3,7,9,12), oviducts (AQPs 1,2,5,8,9) fallopian tubes (AQP 3), uterus (AQPs 1,2,3,4,5, 7,8,9), cervix (AQPs 1,3,4,5,8) and vaginal wall (AQP 1,2,10,12). Extracellular vesicles derived from the female reproductive system have been identified in oviducts, in uterine and vaginal fluids. It is also possible that EV could enter the blood stream via the vasculature of the reproductive system.

Human fallopian tubes have been shown to contain EV [121] suggested to increase fertility, delivering important components to sperm cells. It has been suggested that ‘external’ delivery of such exosomes could act as a potential fertility therapy. The mature egg itself has been shown to release EV, transferring the tetraspanin CD9 to sperm cells aiding capacitation, which is essential for fertilisation [122]. Studies have shown that the introduction of CD9 positive EV allows fertilisation of CD9 negative oocytes by sperm cells. This suggests that administration of CD9 positive exosomes could be used as an infertility treatment [122]. Importantly, EV appear to participate in the cross-talk between maternal and embryo tissues, supporting implantation possibly linked to a role in cell migration [123], [124] a role noted for AQPs in other models and tissues [125].

Endometrial epithelial cell derived EV have been isolated from uterine fluid and characterised [124]. They have been shown possess CD9, also present in the membrane of glandular epithelial cells that express AQP2. To our knowledge no studies have identified AQP2 in UFEV thus far but the functional relevance of AQP2 in EV should be considered. Uterine AQP2 expression is important for implantation and expression of AQP2 & 3 expression is cycle –dependent, increasing at the secretory phase that causes endometrial thickening. It is possible that AQP2 & 3 expression in the endometrium is partially regulated by its removal along with the endometrium, both in whole cells and/or extracellular vesicles.

Exosomes derived from endometrial stem cells have been isolated from menstrual fluid. These exosomes have been shown to improve healing of injured liver tissue in mice [126]. Vaginal exosomes, along with those found in semen [127] and breast milk [128] have been linked to inhibition of HIV-1 viral infection; preliminary studies suggest these exosomes prevent reverse transcription of viral RNA into DNA, during the early stages of infection, therefore preventing its integration into the host genome [129]. Studies characterising EV have not included information on which cell type these are derived from.

Though AQPs have yet to be identified in EV derived from the mammalian female reproductive system, it is evident the AQP-containing cells are producing EV and that AQPs support functions important to successful reproduction. It is likely that AQPs will be present in microvesicles from these cells. Cells containing intravesicular AQPs may release AQPs in exosomes and AQPs from the plasma membrane may also be in exosomes via the endocytic pathway. AQPs are known to increase vesicle membrane stability, in the synthesis of exogenous exosomes in therapeutics AQPs may play an important role in increasing the stability of exosomes, optimising them for delivery.

AQPs 1, 3 and 5 have been identified in human breast tissue [130] where they are believed to play a role in the production of breast milk during lactation. AQP1 is expressed in blood vessels and transports water from the blood stream to the breast tissue. In the human breast AQPs 3 and 5 are expressed by mammary epithelial cells (MECs) which produce breast milk and are in direct contact with breast milk. During late pregnancy and lactation AQP3 is expressed in the cytoplasm of ductal MECs and the basolateral membrane of alveolar MECs. AQP5 relocalised from the cytoplasm of ductal MECs to the apical membrane immediately before parturition, suggesting it plays an important role in the movement of water into breast tissue in the process of generating breast milk [131].

Human breast milk (BM) has been shown to contain EV (BM-EV) that could have potential therapeutic effects against gastrointestinal disease such as necrotizing enterocolitis, a common and serious disease seen in new-born babies that cannot tolerate breastmilk and in those born prematurely where milk supply is not ready. It characterised by inflammation and death of cells in the large intestine [132]. Breast milk exosomes promote intestinal epithelial cell growth and proliferation and stimulate intestinal stem cells. However, they are also likely involved in the involution of the mammary gland post-weaning as they carry MFG-E8 [133] an important factor shown to be necessary for effective involution [134].

Exogenous delivery of breast milk derived exosomes to new-borns who are unable to tolerate breast milk may be a preventative solution against the development of necrotizing enterocolitis [135]. In an in vitro model using rat intestine epithelial cells, BM-EV prevented cell death after exposure to reactive oxygen species (ROS), suggesting an anti-inflammatory role for these EV. This therapy could have wider applications such as, recovery after surgery, Inflammatory Bowel Syndrome (IBS), other gut inflammation diseases. Proteomic analyses of human BM-EV, do not mention AQPs [133], [136], suggesting that if AQPs are present in BM-EV their differential cellular expression levels are not reflected in their EV composition. To our knowledge there are currently no studies that identify AQPs in breast milk exosomes.

To date protein composition studies characterising EV derived from the mammalian male reproductive system have not sought to identify the presence of AQPs [137]. Though these EV has been put forward as important as markers for disease [138]. AQPs are important for successful sperm cell maturation and motility and subsequent fertilisation so it is reasonable to suggest that AQPs could be a biomarker for fertility. Defective AQP3 in sperm cells for example, could easily be detected; sample collection is non-invasive and techniques needed for protein sequence identification quick, easy and inexpensive. If identified as a cause of infertility, AQP3 could be targeted therapeutically. It is known that as sperm cells develop and mature proteins are delivered to them via EV, which they take up; this is likely to include AQPs. Exogenous AQP3-positive EV could be delivered to sperm cells before or during the fertilisation process, which would allow for better osmoregulation by the cells and enhanced sperm cell motility and fertility.

## 5 AQP-EV in the eye

AQP0 plays an important role in lens water permeability and osmotic regulation [139] and may function as a gap junction protein [140]. No studies have yet identified lens fibre-derived microparticles, but Slingsby et al. have hypothesised that EV could explain the intercellular movement of crystallins. These proteins are expressed by lens fibre cells and though they are vulnerable to their oxidative extracellular environment, they have been found in other cells. The group suggests extracellular trafficking of crystallins from lens fibre cells could occur via protective extracellular vesicles [141].

## 6 AQP-EV in the lungs

Lung EV have been identified in a number of pathologies. Tumour-derived lung exosomes have been identified as possible biomarkers for cancer [142] and lung EV are released following tissue injury [143]. It has been shown that alveolar cells make exosomes in response to magnetic iron oxide particles [144]. Though we know that AQP1 is expressed in lung alveoli, AQP1 has not been identified with these EV [145]. The role of cellular AQP1 in the lung remains unclear as the lung environment is near to isoosmolar, suggesting a role alternative to that of a water channel. Without first understanding its cellular role, it is difficult to suggest a potential role for lung–EV AQP1.

AQP4 is also expressed in lung tissue and its expression increases in lung cancer. AQP4 has been shown to be present in fluid collected using this technique. Studies to date do not specify the origin of AQP4 though it may be possible to isolate lung EV using the lung lavage method [146].

## 7 AQP-EV in saliva

Six AQPs have been identified in the digestive system where they play roles in absorption and secretion of water. AQP5 is expressed in salivary glands and is essential in the secretion of water into saliva. Saliva is rich in EV and is thought to be a potential diagnostic tool for a multitude of pathologies. Saliva is important in the digestion of food, lubrication, oral hygiene and taste. AQP5 has been identified in salivary EV secreted by parotid glands [50].

## 8 AQP EV in blood

AQPs have been identified in red and white blood cells. Early descriptions of EV refer to ‘platelet dust’ as microparticles released from platelets were the first to be identified [1].

AQP1 is expressed in reticulocytes (RBCs) where it regulates volumetric changes in response to fluctuations in extracellular osmolality. AQP1 has been identified in exosomes released by maturing RBCs in isotonic environments whereas cells in hypertonic medium retain AQP1 in the plasma membrane [51]. AQP7 has been identified in platelets and is important in the generation of their secretory vesicles. That study suggested that AQP7 could play a role in exosome formation and secretion similar to that of AQP1 in reticulocytes [147].

## 9 Potential roles for AQPs in EV

When considering the roles of EV, the beneficial release of EV for host cells and/or the effects EV have upon recipient cells and their surrounding environment are typically primary considerations. EV are characterised based on their cargo, which can indicate the cell type from which EV are derived and may provide insight to their potential destination and/or function. EV AQP cargo are relevant to the host and recipient cells, but also play roles in EV integrity. Manipulation of the stability of EV may have profound effects on their function by, for example, supporting a longer half-life for the particle which may support more distant intercellular signalling. In cases where the EV may be propagating a detrimental effect such as enhanced inflammation, this may be an important factor in pathogenesis.

Evidence suggests the components of EV are regulated and can be altered by the extracellular environment. EV release can increase or decrease in response to different stimuli and EV cargo and their concentrations can change. The presence of AQP in EV does not appear to be purely as a result of their presence in the plasma membrane. For example, AQP2 loading in uEV is reflective of urine concentration, where lower urine concentration is observed alongside an increase in AQP2 in uEV. AQP1 uEV levels do not increase in AQP1 in hypoosmolar urine though increased cellular expression is observed. The simplest interpretation of this is that AQP presence in EV is regulated beyond their presence in the plasma membrane. Also AQPs 1 and 2 are expressed in different parts of the nephron; AQP1 is expressed in the proximal tubule and AQP2 in the collecting duct. Suggesting more AQP2 is concentrated into uEV in the collecting duct than AQP1 in the proximal tubule.

Overall, AQP EV are found in all body fluids. We suggest that the type and role of the fluid gives some indication of potential EV role and potential role of their cargo. Breast milk, for example, delivers nutrients and immunity to an infant and roles for EV in breast milk could be related to this. However, the EV carriage of proteins such as MFG-E8 will also support breast involution. Urine on the other hand is excreted and removes waste products, suggesting EV and their cargo are expected to be released from system.

### 9.1 AQP EV roles in host cells

#### 9.1.1 Volumetric regulation

**Fig. 4:**
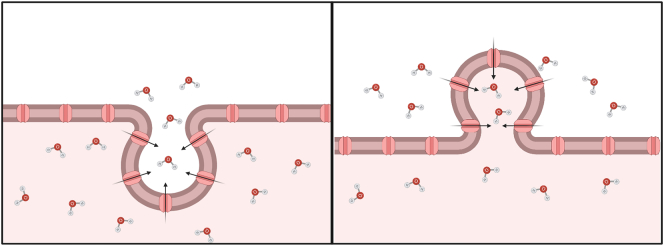
AQPs allow movement of water across the plasma membrane in response to changes in osmotic gradients and membrane AQP concentration determines influx rate. If extracellular hypotonicity is maintained, water influx continues until equilibrium is achieved. Removal of AQPs in EV could reduce membrane water permeability and slow water influx.

Originally it was thought that the main purpose of EV release was one of ‘waste disposal’ to remove unwanted cellular components. Whilst there appears to be support for this notion, it has since been shown that there are other, more complex roles for EV. Nonetheless, the beneficial effects of AQP removal from the plasma membrane may be one explanation for their presence in EV (Fig. 4). A primary role of AQPs in the plasma membrane is that of a water channel allowing movement of water into and out of cells. It has been shown that endogenous regulation of AQPs involves their trafficking to and from the plasma membrane, determining membrane porosity and water permeability. Thus, the concentration of plasma membrane AQPs influences cell volume regulation. In situations where increased levels of plasma membrane AQPs are required, AQP is trafficked from intracellular vesicles, for example AQP2 relocalisation upon vasopressin release [16] or AQP1 relocalisation in response to extracellular hypotonicity in vitro [14]. It is possible that following this trafficking, the plasma membrane and its components are released in EV to the extracellular environment. Depending on AQP function this could decrease water reabsorption or decrease water secretion, features that may be beneficial or detrimental depending upon the local microenvironment. AQP2 is known to play a role in the generation of uterine fluid but for implantation of an egg to occur, uterine fluid levels must be reduced and fluid production decreases. During this time plasma membrane uterine AQP2 levels decrease temporarily; one possible mechanism for the removal of membrane AQP2 is its release into EV. It has been shown that AQP-expressing cells release AQP-containing EV as a mechanism for volumetric control; for example, AQP1 moves into exosomes as red blood cells mature [51]. Also of interest are the effects of volumetric changes on cells; Cho et al. suggest AQP5 relocalisation to the apical membrane following muscarinic receptor activation causes increased water movement into the apical lumen and nuclear shrinkage in parotid cells. This nuclear shrinkage causes the initiation of nuclear processes such as transcription [148]. Considering this, it is possible that removal of AQPs in EV decreases cell volume, helping to initiate transcription pathways. Delivery of secretory AQPs in EV to recipient cells may support volumetric increase and could aid uptake or integration of EV components into the recipient cells.

#### 9.1.2 Vesicle biogenesis

**Fig. 5:**
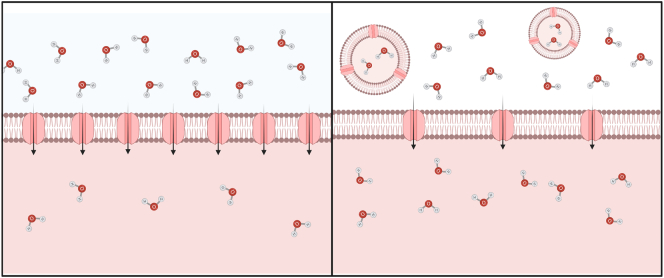
Movement of water through AQPs from the cytoplasm during membrane invagination could allow vesicle biogenesis without increasing intracellular volume. Movement of water from the extracellular space during microvesicle biogenesis from the plasma membrane could increase cell volume locally and facilitate vesicle production.

AQP influence membrane fluidity and dynamics. Increased membrane protein density reduces lipid packing, reducing plasma membrane lipid density. In addition, cells and vesicles that are more water permeable increasing membrane fluidity, which is important in cell motility [149] Increased AQP plasma membrane abundance has been shown to increase cell motility and AQPs can be polarised at the leading edge of motile cells. Although it is not known exactly how AQPs contribute to cell membrane fluidity and dynamics, a number of suggestions has been made. It is possible that during EV biogenesis, both via the endocytic and exocytic pathways, AQPs allow increased localised movement of water through the plasma membrane. During endocytosis AQPs may allow movement of water from the cell, decreasing cell volume and allowing formation and endocytosis of the plasma membrane into the cytoplasm without increasing overall cell volume (Fig. 5). Microvesicles, derived directly from the plasma membrane also contain AQPs ([36], [41], [42]) It is possible that the localised movement of water into the intracellular space increases intracellular volume and aids vesicle formation toward to extracellular space (Fig. 5).

### 9.2 Potential roles in recipient cells of AQP in EV

EV carry cell cargo such as functional DNA, micro RNAs [6], functional proteins [7] enzymes [150], and lipid-based components [3], [4].

#### 9.2.1 Communication – protein delivery

**Fig. 6:**
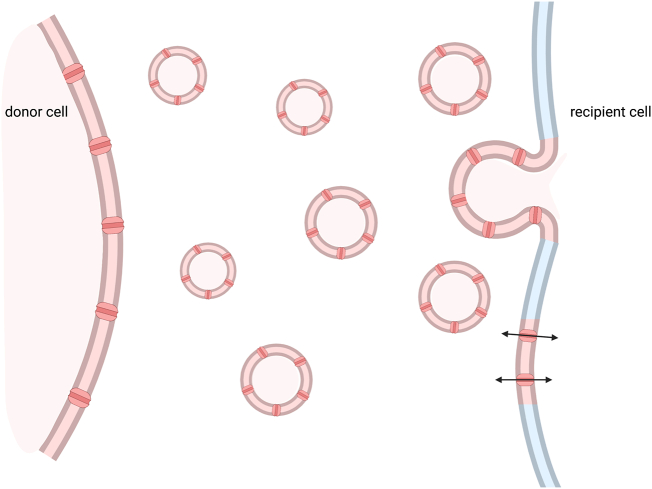
AQP positive donor cells release AQP positive EV. These EV move through the extracellular environment and can be taken up and integrated into recipient cells. This is a method by which functional proteins can be transferred between cells. EV transported AQPs act as functional water channels in recipient cells [35].

A major role for EV is intercellular communication. It has been shown that EV cargo are reflective of the state and health of the donor cell. EV and their cargo share information with recipient cells and in response, recipient cells are altered. Street et al. have shown that AQP proteins can be transported to neighbouring cells via EV and may be taken up and function in these cells [35]. As such, we speculate a potential role for AQPs in cell to cell communication where AQPs are delivered to AQP negative cells via EV (Fig. 6). Increased levels of AQP in EV could signal to neighbouring cells, increasing AQP expression. In considering the potential physiological benefits of this mechanism, the rate at which EV are generated and released compared to the time it takes for AQP proteins to be synthesised using transcription could be an important consideration. EV biogenesis and delivery to recipient cells is a quick process, requiring less from the recipient cell. AQP sharing via EV could therefore be a rapid solution for cells requiring increased AQP levels.

#### 9.2.2 Communication – bystander effect

One interesting aspect of EV is the ability to propagate a phenotype to neighbouring cells – a feature termed the ‘bystander effect’. This has been noted in cases where irradiated cells can transmit a radiation-damage phenotype to non-irradiated cells [151]. This has also been shown in the case of heat-shock and raises the tantalising possibility that insult to a cell can lead to increased preparedness for that insult in neighbouring cells [152]. If this is coupled to a greater half-life of the EV, this may permit a protective response in more distant cells. A similar role may be possible for AQP in EV in intercellular communication where presence of AQP in EV cause a bystander Effect, signalling other AQP positive cells to increase or decrease AQP expression.

#### 9.2.3 Communication – inflammation

It is clear that EV can have profound impacts on immune function and inflammatory responses but, depending on the context and the cargo they carry, they can be pro- or anti- inflammatory [8]. In different settings, EV can be active in recruiting macrophages [153] an important innate immune cell capable of both pro-and anti-inflammatory responses as well as modifying those responses. The control of this response is critical in resolution of inflammatory responses and promoting wound healing [154]. AQPs have also been implicated as important molecules in a number of immune processes such as phagocytic cup formation [155] and immune cell migration [149]. This raises the possibility that AQP carriage in EV may mediate these important effects. AQP1 has been evidenced as crucial in the process of macrophage phenotype switching that determines their inflammatory properties. Though to our knowledge no studies have yet ascertained the inflammatory role of AQPs in EV and their impact on macrophage phenotype, their increased concentration in circulating EV following injury suggests this is an interesting and important area of research. Of note is the suggestion that EV from GBM can modulate microglial phenotypes and support tumour growth [156], [157]. Our unpublished work has identified the presence of AQP in EV released from resected GBM. Further work is needed to bring together these strands to a more complete view of the role of EV in different contexts and their impact on inflammatory responses, which can be both protective and damaging, depending on the precise control in different circumstances.

#### 9.2.4 Communication- protein orientation

**Fig. 7:**
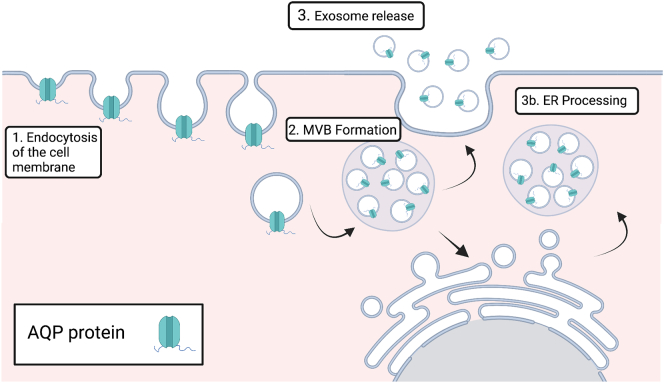
It has been suggested that membrane protein orientation can change in EV with proteins appearing to be ‘inside out’. This could occur through the processing of MVB in the endoplasmic reticulum. If AQPs change orientation during this process, their N- and C- termini would be exposed to the extracellular/extravesicular environment.

It has been shown that the process of endocytosis of the plasma membrane and its release as exosomes can cause orientation switching of membrane proteins [54]. In the context of AQPs, this would mean that the normally intracellular N- and C termini and intracellular loops would be exposed to the extracellular/extra-vesicular space (Fig. 7). These regions contain phosphorylation sites and regions important in protein interactions [72].

### 9.3 Potential roles of AQP in EV – in EV delivery

#### 9.3.1 Membrane integrity and volumetric regulation

Upon release from the host cell, EV journey through fluid environments with differing osmolality to their host cell. These environments may be prone to extreme fluctuations in osmolality and EV must be able to adapt to these in order to maintain their integrity. AQP positive cells are able to regulate membrane integrity and we anticipate AQP will pay a similar role in EV, preventing rapid swelling or shrinkage in response to environmental changes, as seen for example in sperm cells [158]. Shrinking or swelling of EV could result in unsuccessful delivery of EV cargo to recipient cells and subsequent release of cargo into the extracellular space may have undesired consequences such as inflammation. AQP1 moves into exosomes as RBCs mature, suggesting a role for volumetric control [51]. This raises the possibility that the role of AQPs in helping to maintain volumetric control in EV may underpin EV resilience to environmental change and, consequently, their half-life. This will enable longer journeys within the body to impact more distant recipient cells and/or enter more distant sites for sampling as biomarkers.

#### 9.3.2 EV deformability

Lenzini et al. observe that in order to travel through the extracellular environment, specifically the extracellular matrix, EV must be able to fit through spaces smaller than EV diameter. They hypothesise that EV must have the ability to deform as they have been found in locations distant from their origin [52]. They show that AQP1 positive EV have the ability to move through small spaces more easily that those with reduced functional AQP1. The group suggests the water permeability of EV dictates their level of deformability though volumetric regulation. As deformability is required for EV to move though small spaces, presence of AQPs in EV could be essential for successful migration of EV. It is also likely that the increased membrane fluidity of AQP positive EV contributes to EV ability to deform.

#### 9.3.3 EV-recipient cell fusion

Kelly et al. suggest there could be an important role for AQPs in the generation and release of vesicles, specifically for vesicle swelling which facilitates their fusion with the plasma membrane [159]. The authors of that study demonstrated that AQP-positive EV were able to swell and fuse with the plasma membrane, subsequently releasing their secretory components. This same concept could be applied to the fusion of circulating EV with the plasma membrane of recipient cells where AQP-positive EV have the ability to swell in response to osmolar changes near the plasma membrane, aiding fusion and allowing uptake of EV by recipient cells and therefore facilitating integration of EV cargo.

## 10 AQP miRNA and EV

EV are well established to carry miRNA and that this can be important in the function of those EV. For example, the carriage of miRNA from EV derived from GBM, can drive macrophage (microglial) reprogramming as a result of miR-21 and others [156]. Such miRNA can have profound regulatory effects on microglia and thus inflammatory responses [160]. However, this principle may extend further, to EV-mediated control of AQP, possibly as a route to mediating a bystander protective effect in anticipation of an osmotic stress.

It has been shown that miR320a can reduce expression of AQP1 and 4, both in vitro and in vivo. More specifically though, miR-130a transcriptionally reduces the M1 isoform of AQP-4, a level of control that can be reversed with the use of anti-miR-130a [161].

## 11 AQPs in EV as biomarkers for disease

Changes in AQP expression are seen in many pathologies. This includes both cellular and EV expression. Pathologies are varied and include cancers, tissue injury, ischemia, inflammatory disease. These differences in AQP expression can often be measured and can be used as biomarkers for disease. Clinical measurement of the cellular expression levels of AQPs can be invasive as sample collection may include tissue biopsy, CSF collection or blood sampling. In contrast, extraction of fluids containing EV are often less invasive, for example if EVs can be collected from saliva or urine. Further research into AQP concentration and function in EV could enhance clinical diagnostic procedures.

## 12 EV-AQPs as drug targets

As AQPs have been implicated in various pathologies, they are considered important potential drug targets. To date, proposed inhibitors of AQPs have proved largely unsuccessful at clinical trial. As such a number of studies have sought to target AQPs using siRNAs in EV that target and alter AQP expression [162], [163]. This is a potentially promising area for AQP targeted therapeutics.

## 13 AQPs in EV drug delivery

It has been shown that endogenous EV can have therapeutic effects on injured tissue in different organisms, promoting wound healing. As such, EV as therapeutics has become an exciting new area of research [164]. EV can be extracted from the appropriate fluid, purified and administered to the affected individual. Understanding active components of EV will advance this area further, allowing exogenous synthesis of EV, reducing the need for host organisms. As EV membrane composition is important for the successful delivery of EV in plants [23] we hypothesise AQP would be essential components of synthesised EV membranes.

## 14 Conclusions

The study of EV is an exciting and still emerging area of research in intercellular communication. It is clear that EV have profound impacts on both inflammatory and repair responses and have important roles to play in health and disease, including those scenarios where AQPs also have an important role. Understanding the role of AQPs within extracellular vesicles is still in its infancy and further studies are needed to fully define the presence of AQPs as well as their function in these different scenarios. There is much work to be undertaken to fully understand this important interaction and especially to define the functional significance of AQPs within EVs in different locations as they journey from donor to recipient cell.

## Declaration of competing interest

The authors declare that they have no known competing financial interests or personal relationships that could have appeared to influence the work reported in this paper.
